# Characteristics and Prediction Accuracy According to Corneal Stiffness in Suspected Keratoconus

**DOI:** 10.3390/jcm15124577

**Published:** 2026-06-12

**Authors:** Se Hoon Choi, Seung Hyen Lee, Hyun Sung Leem

**Affiliations:** 1 Department of Optometry, Eulji University College of Health Science, Seongnam 13135, Republic of Korea; jake5451@hanmail.net; 2 Department of Ophthalmology, Eulji University College of Medicine, Nowon Eulji University Hospital, Seoul 01830, Republic of Korea

**Keywords:** keratoconus, corneal stiffness, biomechanics

## Abstract

**Background/Objectives**: This study aimed to evaluate the biomechanical characteristics of the cornea to assess their diagnostic accuracy in distinguishing normal eyes from those suspected keratoconus eyes. **Methods**: In this cross-sectional study, corneal elevation and curvature radius were measured in 217 participants using Pentacam. Average values were obtained based on the best-fit sphere (BFS) and the enhanced best-fit sphere (EBFS). The biomechanical characteristics of the cornea were assessed using the Corvis ST device. Receiver operating characteristic curve analysis was performed to determine the diagnostic accuracy. **Results**: The radii of the BFS in the anterior and posterior corneas were significantly larger in the normal group compared to the suspected keratoconus group. Conversely, EBFS elevation values in both the anterior and posterior corneas were lower in the normal group. The velocity at which the cornea was first flattened had the highest diagnostic accuracy for identifying suspected keratoconus. **Conclusions**: Eyes with suspected keratoconus had a significantly smaller corneal radius on both the anterior and posterior surfaces compared with normal eyes. In addition, due to the increased deformability and reduced resistance to a given force, these parameters serve as valuable biometric indicators for distinguishing suspect eyes from normal eyes.

## 1. Introduction

The refractive power of the cornea is approximately +43.05 D, which accounts for two-thirds of the total refractive power of the human eye, and therefore plays a crucial role in vision. The stromal layer of the cornea comprises approximately 90% of its thickness, and it is known that the regular arrangement of collagen fibers is essential for maintaining corneal transparency. Stiffness refers to the degree to which a material resists deformation when an external force is applied. In the cornea, this mechanical property is primarily influenced by the density and thickness of its collagen fibers [1].

Keratoconus is a progressive ectatic disorder characterized by thinning and anterior protrusion of the cornea due to structural alterations in collagen structure. Although the exact etiology of keratoconus remains unclear, keratoconus is fundamentally associated with a reduction in corneal stiffness (mechanical strength). There is a continuous decline in corneal stiffness index values corresponding to an increase in the severity of keratoconus. Given that reduced corneal rigidity is the key pathophysiology of keratoconus, the major of treatment is to restore or reinforce its stiffness. Corneal Collagen Cross-Linking (CXL), which is performed to stop the progression of keratoconus, works by increasing mechanical rigidity [2,3,4].

**Figure 1:**
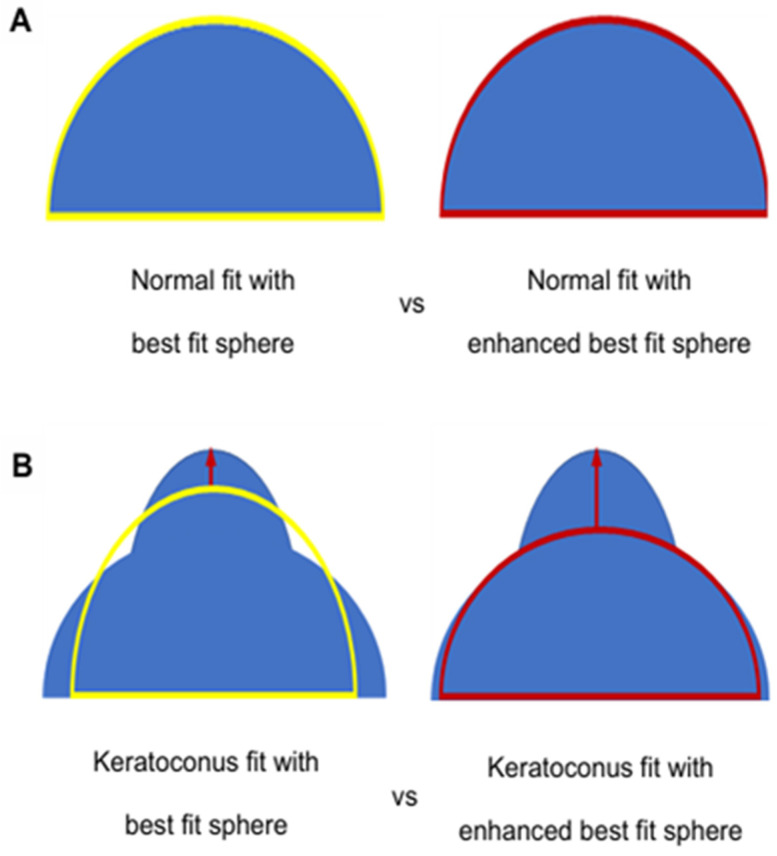
(**A**) Schematic comparison of normal cornea showing minimal difference in elevation between the standard BFS (yellow) and EBFS (red), indicating a nearly spherical shape. (**B**) Schematic comparison of keratoconic cornea showing the significant increase in apical elevation when using the EBFS (red) compared to the standard BFS (yellow). Red arrows indicate the elevation difference between the reference spheres and the corneal apex.

The best-fit sphere (BFS), which compares the relative heights at the central 9 mm of the cornea, is useful for diagnosing keratoconus. The enhanced best-fit sphere (EBFS), derived from measurements of all areas except for a 4 mm radius centered on the thinnest part of the cornea, sets a more appropriate reference sphere for peripheral parts and thus distinctly demonstrates corneal ectasia (Figure 1). BFS and EBFS values indicate morphological deformation of the cornea and serve as indirect indicators of reduced corneal stiffness. While advanced keratoconus can be easily identified using corneal topography, accurate diagnosis and prevention at suspected stages remain challenging. Biomechanical instability of the cornea may precede detectable topographical changes. Therefore, early recognition of biomechanical instability in the cornea could play a crucial role in achieving accurate early diagnosis [5,6,7,8].

The research was conducted based on the hypothesis that corneal biomechanical weakening occurs before measurable topographical changes. Therefore, this study aimed to compare elevation and radius values in both the anterior and posterior surfaces between normal eyes and eyes with suspected keratoconus using BFS and EBFS. In addition, we also sought to analyze the biomechanical characteristics of the cornea to determine diagnostic accuracy in identifying suspected keratoconus.

## 2. Materials and Methods

This research was conducted prospectively, and all participants were enrolled only after they verbally and in writing agreed with the purpose and methods of the study and provided their informed consent. All study procedures were approved by the Eulji University Institutional Review Board (Approval No.: EU22-082) before testing and all procedures.

### 2.1. Patient Criteria

The normal group included healthy adults with normal findings on slit-lamp examination and corneal topography, who had no history of ophthalmic surgery or systemic diseases that could affect the eye. The suspected keratoconus group comprised subjects who met all the following Pentacam HR (Oculus, Wetzlar, Germany) criteria without any definitive clinical signs on slit-lamp examination (such as Fleischer’s ring or Vogt’s striae): Sim-K astigmatism ≥ 1.50 D, inferior–superior corneal asymmetry value (I–S value) ≥ 1.20 D, central corneal refractive power ≥ 47.00 D, and corneal thickness < 500 μm. Subjects were excluded from this group if they had advanced Pentacam parameters (central corneal refractive power ≥ 48.70 D or I–S values ≥ 1.90 D), or had any ophthalmic surgical history or ocular trauma [9,10]. The study excluded individuals aged between their 10s and 40s who had corneal diseases, systemic diseases that could affect the cornea, or had undergone laser refractive surgery.

### 2.2. Measurements

All measurements were performed three times and the average values were used for analysis. Corneal refractive power was obtained as the mean refractive power of the principal meridians measured using the Pentacam. The elevations of both the anterior and posterior surfaces of the cornea were measured by taking the average value of anterior ectasia at the thinnest point based on both BFS and EBFS. The radii of both the anterior and posterior corneal surfaces were calculated as the mean values based on the BFS and EBFS using the Pentacam.

Corneal deformation amplitude was assessed using Corvis ST (Oculus Optikgeräte GmbH, Wetzlar, Germany), which employs a high-speed Scheimpflug camera to record the dynamic corneal response to an air puff. The following parameters were obtained: time to first (A1T) and second (A2T) applanation, length flattened (A1L, A2L), and velocity (A1V, A2V) after a puff of air pressure at a constant force. Additionally, measurements were made for average values, such as the time to maximum deformation (HCT), curvature (HCR), distance between points of inflection (HCPD), and degree of deflection amplitude (HCPD) when the cornea was deformed to its maximum extent.

### 2.3. Statistical Analysis

All statistical analyses were performed using the statistical software SPSS (version 21.0; SPSS Inc., Chicago, IL, USA). To adjust for the inter-eye correlation resulting from the inclusion of multiple eyes from the same participant, Generalized Estimating Equations (GEEs) with an exchangeable working correlation matrix were applied to compare continuous clinical variables between the two groups, replacing the standard independent *t*-test. To determine the accuracy of screening diagnostic tests between normal and suspected keratoconus groups, receiver operating characteristic (ROC) curve analysis was used to calculate the area under the curve (AUC). If the AUC was above 0.80, it was considered excellent; if it was between 0.70 and 0.80, it was considered good; and if it fell below 0.70, it was deemed inappropriate [11]. A *p*-value of *p* < 0.05 was regarded as statistically significant.

## 3. Results

A total of 217 participants were enrolled, comprising 131 participants (201 eyes) in the normal group and 86 participants (130 eyes) in the suspected keratoconus group. The average ages of the two groups were 26.96 ± 5.10 years and 27.70 ± 5.07 years, respectively (Table 1).

### 3.1. Corneal Radius and Elevation Parameters

The radius of the BFS on the anterior corneal surface was significantly larger in the normal group than in the suspected keratoconus group (*p* < 0.001). Similarly, the radius of the EBFS was also significantly larger in the normal group than in the suspected keratoconus group (*p* < 0.001). Moreover, there was a significantly smaller difference in the radius due to sphere enhancement in the normal group than in the suspected keratoconus group (*p* = 0.007). The anterior displacement at the thinnest point of the cornea relative to the BFS was significantly larger in the suspected keratoconus group than in the normal group (*p* < 0.001), and was also significantly larger for suspected keratoconus than for EBFS (*p* < 0.001). Furthermore, the change in anterior displacement due to sphere enhancement was significantly larger in the suspected keratoconus than in the normal group (*p* < 0.001) (Table 2).

On the posterior corneal surface, the radius of the BFS was significantly larger in the normal group than in the suspected keratoconus group (*p* < 0.001), and the radius of the EBFS was significantly larger in the normal group (*p* < 0.001). The anterior displacement at the thinnest part of the cornea relative to the BFS on the posterior corneal surface was significantly greater in the suspected keratoconus group than in the normal group (*p* < 0.001), and was also significantly greater for suspected keratoconus than for EBFS (*p* < 0.001). Furthermore, there was a significantly larger difference in anterior displacement due to sphere enhancement within the suspected keratoconus group than in the normal group (*p* = 0.015) (Table 3).

### 3.2. Cornea Deformation Amplitude Parameters

The time taken for the cornea to first flatten (A1T) when air was blown onto the cornea and then pushed back was significantly longer in the normal group than in the suspected keratoconus group (*p* < 0.001), and the speed (A1V) was significantly slower in the normal group than in the suspected keratoconus group (*p* < 0.001). Additionally, the flattening length (A1L) was significantly longer in the normal group (*p* < 0.001). During the process where the cornea was pushed back and returned to its original position, when it flattened for a second time (A2T), it took significantly longer for those with suspected keratoconus than those in the normal group (*p* = 0.001), and the speed (A2V) of this action was also faster (*p* < 0.001). Moreover, the length (A2L) of action was significantly greater in normal subjects than in those with suspected keratoconus (*p* < 0.001). The deflection amplitude, which is the difference between the extent to which the cornea is pushed back at maximum deformation and total eye movement (HCDA), was significantly greater among those with suspected keratoconus compared to those from the normal group (*p* < 0.001), while the radius of curvature (HCR) at that point was smaller among those with suspected keratoconus than that of the normal group (*p* < 0.001) (Table 4).

### 3.3. Diagnostic Accuracy

When the anterior displacement threshold for BFS was set at 3.50 μm, the sensitivity and specificity for diagnosing suspected keratoconus were 50.0% and 97.0%, respectively. The area under the ROC curve (AUC) for the diagnosis of suspected keratoconus was good (0.776). For the EBFS, an anterior displacement threshold of 7.50 μm yielded a sensitivity of 46.9% and specificity of 94.0%, with a similarly good AUC of 0.742. When the anterior displacement criterion was 5.50 μm for BFS, diagnostic sensitivity and specificity were 65.4% and 63.2%, respectively. At this threshold, the AUC for the diagnosis of suspected keratoconus was rated as good, with a value of 0.712.

**Figure 2:**
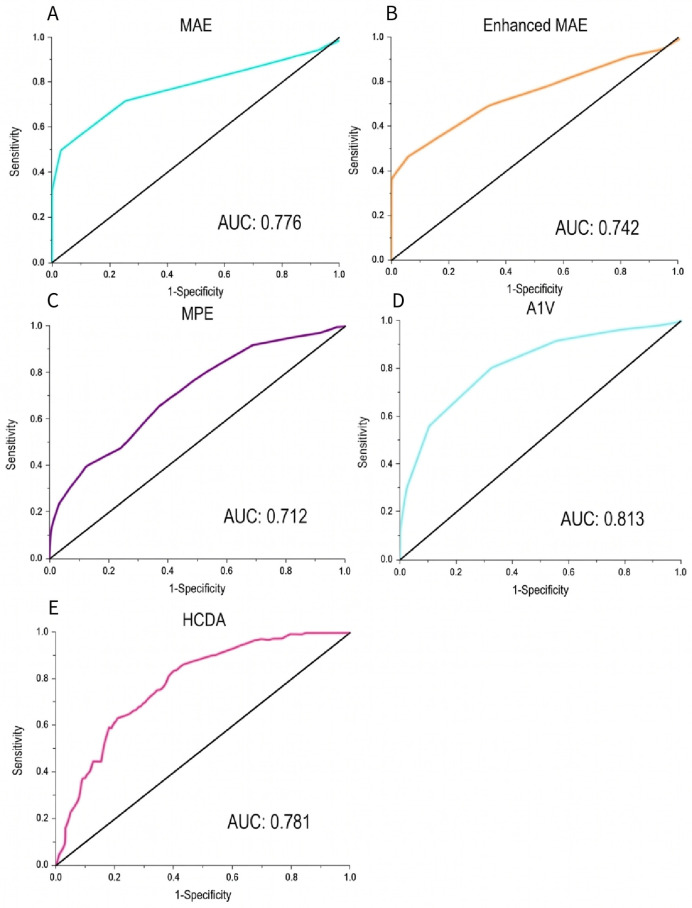
ROC curves and AUC to separate normal and keratoconus suspect group. (**A**) MAE (AUC = 0.776), (**B**) enhanced MAE (AUC = 0.742), (**C**) MPE (AUC = 0.712), (**D**) A1V (AUC = 0.813), (**E**) HCDA (AUC = 0.781).

When the criterion for the speed at which the cornea first flattens was set at 0.165 m/s, the sensitivity and specificity for diagnosing suspected keratoconus were 80.0% and 67.7%, respectively. At this point, the standalone AUC for identifying suspected keratoconus was modest yet acceptable at 0.813. When the threshold for deflection amplitude, which is the difference between the extent to which the cornea is pushed back at maximum deformation and total eye movement, was set at 1.055 mm, the sensitivity and specificity for diagnosing suspected keratoconus were 81.5% and 61.7%, respectively. At this point, the AUC for diagnosing suspected keratoconus was determined to be good at 0.781 (Figure 2).

## 4. Discussion

This study highlights the significance of corneal biomechanical assessment in distinguishing suspect eyes from normal eyes. Biochemical parameters such as A1T and HCDA, which demonstrated high diagnostic accuracy, indicate that subtle morphological changes caused by reduced corneal stiffness can already be detected during the suspected stage, providing valuable complementary insights alongside standard topographic screenings.

Miháltz et al. [5] and Samira et al. [12] reported results consistent with our study, finding in their corneal topography analysis using Pentacam that both the anterior displacement of the front surface of the cornea and the anterior displacement of the back surface of the cornea were significantly greater in the keratoconus and suspected keratoconus groups than in normal eyes. Fam et al. [13] and Sonmez et al. [14] emphasized the diagnostic importance of posterior corneal elevation, showing higher anterior displacement of the posterior cornea in eyes with keratoconus and suspected keratoconus compared to normal eyes, in agreement with our results. In addition, Toprak et al. [15] and Kanclerz et al. [16] also highlighted the importance of anterior displacement on the posterior surface of the cornea for diagnosing keratoconus, and reported higher anterior displacements in this area than in normal eyes in cases of keratoconus and its progression, findings that align with our research.

Consistent with our findings, Schlegel et al. [9] and Lee et al. [17] reported that the radii of both BFS and EBFS on anterior and posterior surfaces were significantly smaller in eyes with suspected keratoconus compared with normal eyes. Similarly, Gatinel et al. [18] suggested that anterior displacement of the cornea influences changes in the radius of the BFS during topographic analysis, which aligns with our results. These results may be explained by the progressive nature of keratoconus, in which forward protrusion of the cornea leads to morphological alterations on both the anterior and posterior surfaces, even in eyes with suspected stage of the disease.

Previous comparative studies [19,20] using the Corvis system have demonstrated that the time, speed, and length parameters associated with corneal flattening were consistent with the result of the present study. Furthermore, the deflection amplitude at maximum corneal deformation was significantly smaller in the normal group than in the suspected keratoconus group, whereas the radius of curvature was significantly larger in the normal group. These results also support that eyes with suspected keratoconus exhibit greater deformability and reduced stiffness compared with normal eyes.

In our study, A1V demonstrated the highest diagnostic accuracy (AUC = 0.813) in differentiating normal eyes from keratoconus suspects. While posterior elevation changes (such as MPE and Enhanced MAE) have traditionally been considered key tomographic signs of early ectasia, our findings indicate that an explicit biomechanical alteration can be detected acutely through A1V.

This can be explained by the underlying pathophysiology of early keratoconus. The disease process begins with micro-structural modifications, including the slippage of collagen fibrils and the extracellular substance and loss of inter-lamellar cohesion, which compromises the cornea’s overall stiffness. When exposed to a constant air-puff force, this early reduction in mechanical resistance allows the cornea to deform more rapidly during the initial phase. Consequently, A1V captures this immediate kinetic response before substantial focal thinning or macroscopic posterior protrusion manifests topographically, highlighting its superior performance as an early predictive marker.

Biomechanically normal corneas tend to require more time to be flattened and have a slower speed. Similarly, corneas with normal structural integrity and viscoelasticity generally show smaller deflection amplitudes, resulting in a larger radius of curvature. Conversely, corneas with distorted structure and reduced viscoelasticity are more easily deformed under a given force, demonstrating greater deflection amplitude and, consequently, a smaller radius of curvature. No significant differences were observed between the normal and suspected keratoconus groups in the time taken to reach maximum deformation and the distance between inflection points. These findings were consistent with previously reported findings [21,22,23,24].

We critically consider the small temporal differences between groups, specifically 0.14 ms for A1T and 0.13 ms for A2T. Given that the Corvis ST’s ultra-high-speed camera (4330 frames/s) yields a temporal resolution of approximately 0.23 ms per frame, these minute differences fall slightly below the single-frame sampling baseline and could be influenced by measurement noise. However, because our data represent the mathematical means of pooled eyes across a large cohort, these subtle yet consistent shifts achieve high statistical significance under GEE modeling. There were some limitations to this study. Although it was conducted prospectively, the final comparison between the two groups and the derivation of the cut-off values were based on a single-point cross-sectional comparison. Therefore, the present cross-sectional study design does not possess longitudinal follow-up data to monitor actual disease progression over time or to map longitudinal forecasting. Further longitudinal research is needed to validate these findings and clarify their predictive value for keratoconus progression. Another limitation of this study is that all participants were Korean, which may restrict the applicability of the results to other ethnic groups. Further multicenter studies involving diverse populations are necessary to validate the universality of these corneal biomechanical parameters.

## 5. Conclusions

In conclusion, eyes with suspected keratoconus exhibited a significantly reduced radius and increased anterior displacement on both the anterior and posterior corneal surfaces relative to the BFS and EBFS compared to normal eyes. Among the biomechanical parameters, the first applanation velocity (A1V) showed the highest diagnostic accuracy for identifying suspected keratoconus. Therefore, rather than serving as a definitive standalone diagnostic tool, a comprehensive evaluation of both topographical parameters and corneal deformation amplitude serves as a supportive screening aid that offers complementary diagnostic value in identifying suspected keratoconus.

## Figures and Tables

**Table 1:** The clinical characteristics of subjects.

	Normal	Keratoconus Suspect	*p* Value
Patients (*n*)	131 (65 M, 66 F)	86 (20 M, 66 F)	<0.001
Age (years)	26.96 ± 5.10	27.70 ± 5.07	0.402
SE (Diopter)	−4.49 ± 2.22	−6.20 ± 2.77	<0.001
K1 (Diopter)	42.22 ± 1.21	44.65 ± 0.93	<0.001
K2 (Diopter)	43.20 ± 1.27	47.19 ± 0.83	<0.001
TP (μm)	557.90 ± 27.32	485.62 ± 13.11	<0.001

All values are expressed as mean ± standard deviation. SE, spherical equivalent; TP, thinnest pachymetry. M, male; F, female.

**Table 2:** Comparison of the mean value of the anterior corneal radius and elevation with a Pentacam.

	Normal	Keratoconus Suspect	*p* Value
BFS radius (mm)	7.98 ± 0.23	7.46 ± 0.13	<0.001
Enhanced BFS radius (mm)	8.01 ± 0.23	7.50 ± 0.13	<0.001
BFS change (mm)	0.03 ± 0.01	0.03 ± 0.02	0.014
MAE (μm)	1.87 ± 0.97	3.53 ± 1.99	<0.001
Enhanced MAE (μm)	4.54 ± 1.97	7.18 ± 3.48	<0.001
Elevation change (μm)	2.68 ± 1.19	3.65 ± 1.73	<0.001

All values are expressed as mean ± standard deviation. BFS, best-fit sphere; MAE, maximal anterior elevation on thinnest point.

**Table 3:** Comparison of the mean value of the posterior corneal radius and elevation with a Pentacam.

	Normal	Keratoconus Suspect	*p* Value
BFS radius (mm)	6.49 ± 0.21	6.10 ± 0.14	<0.001
Enhanced BFS radius (mm)	6.53 ± 0.21	6.14 ± 0.15	<0.001
BFS change (mm)	0.04 ± 0.02	0.04 ± 0.02	0.768
MPE (μm)	4.72 ± 2.50	7.39 ± 3.90	<0.001
Enhanced MPE (μm)	10.21 ± 4.95	13.75 ± 6.78	<0.001
Elevation change (μm)	5.50 ± 2.90	6.35 ± 3.41	0.029

All values are expressed as mean ± standard deviation. BFS, best-fit sphere; MPE, maximal posterior elevation on thinnest point.

**Table 4:** Comparison of the mean value of the cornea with a Corvis.

	Normal	Keratoconus Suspect	*p* Value
A1T (ms)	7.62 ± 0.25	7.48 ± 0.20	<0.001
A1V (m/s)	0.16 ± 0.02	0.18 ± 0.02	<0.001
A1L (mm)	2.27 ± 0.35	1.98 ± 0.34	<0.001
A2T (ms)	21.89 ± 0.31	22.02 ± 0.35	0.003
A2V (m/s)	−0.28 ± 0.03	−0.30 ± 0.03	<0.001
A2L (mm)	1.97 ± 0.36	1.60 ± 0.33	<0.001
HCDA (mm)	1.04 ± 0.09	1.13 ± 0.08	<0.001
HCR (mm)	7.50 ± 0.81	6.39 ± 0.67	<0.001

All values are expressed as mean ± standard deviation. A1T, first applanation time; A1V, first applanation velocity; A1L, first applanation length; A2T, second applanation time; A2V, second applanation velocity; A2L, second applanation length; HCDA, highest concavity deformation amplitude; HCR, highest concavity radius.

## Data Availability

The data that support the findings of this study are available from the corresponding author upon reasonable request.

## Abbreviations

The following abbreviations are used in this manuscript:

BFS	Best-fit sphere
EBFS	Enhanced best-fit sphere
MAE	Maximal anterior elevation on thinnest point
MPE	Maximal posterior elevation on thinnest point
A1T	First applanation time
A1V	First applanation velocity
A1L	First applanation length
A2T	Second applanation time
A2V	Second applanation velocity
A2L	Second applanation length
HCDA	Highest concavity deformation amplitude
HCR	Highest concavity radius
HCPD	Highest concavity peak distance
HCT	Highest concavity time
